# Supplementary material for: Subversion of Host Cell Mitochondria by RSV to Favor Virus Production is Dependent on Inhibition of Mitochondrial Complex I and ROS Generation
Source: Cells. 2019 Nov 11;8(11):1417. doi: 10.3390/cells8111417 (PMC6912631; doi:10.3390/cells8111417)
Supplement: Supplementary File 1 [file cells-08-01417-s001.pdf]

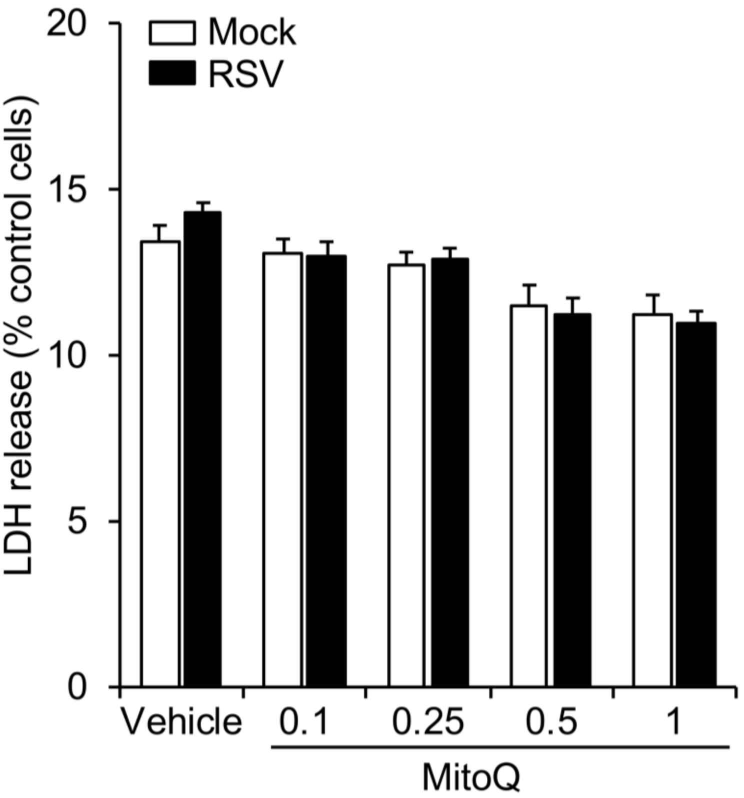

Figure S1. Lack of effect of MitoQ dose-response treatments on cell viability. A cytotoxicity detection kit (LDH Release Assay, Roche Applied Science) was used to analyse cell death on the basis of the amount of LDH (lactate dehydrogenase) released into the medium upon plasma membrane damage. A549 cells cultured in 96-well plates were infected with RSV (MOI 1, 24 h) with the addition of DMSO (vehicle) or MitoQ (0.1, 0.25, 0.5, and 1 μM) for the last 16 h. The LDH assay was then carried out as previously [19, 23]. Results represent the mean + SEM (n = 3).
